# Supplementary material for: Can digital skill protect against job displacement risk caused by artificial intelligence? Empirical evidence from 701 detailed occupations
Source: PLoS One. 2022 Nov 8;17(11):e0277280. doi: 10.1371/journal.pone.0277280 (PMC9642882; doi:10.1371/journal.pone.0277280)
Supplement: S1 Table — (DOCX) [file pone.0277280.s001.docx]

**S1 Table. The full list of estimation results.**

| **Major Occupational Categories** | **log of Wage** | **log of Employment** |
| --- | --- | --- |
| 1.Architecture and Engineering (N=238) | −0.0058*** | −0.0054 |
|  | (0.0004) | (0.0036) |
| 2.Arts, Design, Entertainment, Sports, and Media (N=231) | −0.0027*** | −0.0066* |
|  | (0.0006) | (0.0035) |
| 3.Building and Grounds Cleaning and Maintenance (N=56) | −0.0013*** | −0.0190 |
|  | (0.0026) | (0.0207) |
| 4.Business and Financial Operations (N=210) | −0.0020*** | −0.0088** |
|  | (0.0005) | (0.0039) |
| 5.Community and Social Service (N=91) | −0.0006 | 0.0126 |
|  | (0.0029) | (0.0277) |
| 6.Computer and Mathematical (N=119) | −0.0049*** | −0.0213** |
|  | (0.0005) | (0.0097) |
| 7.Construction and Extraction (N=392) | −0.0047*** | −0.0156*** |
|  | (0.0006) | (0.0059) |
| 8.Educational Instruction and Library (N=154) | −0.0033*** | −0.0155** |
|  | (0.0007) | (0.0060) |
| 9.Farming, Fishing, and Forestry (N=77) | 0.0024 | −0.0250** |
|  | (0.0025) | (0.0100) |
| 10.Food Preparation and Serving Related (N=112) | −0.0070*** | 0.0372*** |
|  | (0.0009) | (0.0114) |
| 11.Healthcare Practitioners and Technical (N=308) | −0.0070*** | 0.0117*** |
|  | (0.0008) | (0.0043) |
| 12.Healthcare Support (N=98) | −0.0041*** | −0.0040 |
|  | (0.0010) | (0.0067) |
| 13.Installation, Maintenance, and Repair (N=350) | −0.0037*** | −0.0149*** |
|  | (0.0005) | (0.0046) |
| 14.Legal (N=56) | −0.0064*** | 0.0036 |
|  | (0.0011) | (0.0094) |
| 15.Life, Physical, and Social Science (N=294) | −0.0041*** | 0.0048* |
|  | (0.0006) | (0.0028) |
| 16.Management (N=217) | −0.0021** | −0.0028 |
|  | (0.0008) | (0.0047) |
| 17.Office and Administrative Support (N=364) | −0.0024*** | −0.0002 |
|  | (0.0005) | (0.0064) |
| 18.Personal Care and Service (N=210) | −0.0038*** | −0.0009 |
|  | (0.0007) | (0.0042) |
| 19.Production (N=721) | −0.0029*** | −0.0038 |
|  | (0.0005) | (0.0042) |
| 20.Protective Service (N=133) | −0.0067*** | −0.0003 |
|  | (0.0008) | (0.0065) |
| 21.Sales and Related (N=140) | −0.0075*** | −0.0046 |
|  | (0.0008) | (0.0052) |
| 22.Transportation and Material Moving (N=336) | −0.0047*** | −0.0005 |
|  | (0.0008) | (0.0038) |

Note: *** *p* < 0.01, ** *p* < 0.05, and * *p* < 0.1; the standard error is shown in parentheses under the coefficient.
