# Supplementary material for: Can digital skill protect against job displacement risk caused by artificial intelligence? Empirical evidence from 701 detailed occupations
Source: PLoS One. 2022 Nov 8;17(11):e0277280. doi: 10.1371/journal.pone.0277280 (PMC9642882; doi:10.1371/journal.pone.0277280)
Supplement: S2 Table — (DOCX) [file pone.0277280.s002.docx]

**S2 Table. The full list of alternative measurements.**

| **Sectors** | **Code** | **Detailed Occupations** | **Risk_d** |
| --- | --- | --- | --- |
| Accommodation and food services | 35-3041 | Food Servers; Nonrestaurant | 0.73 |
|  | 35-2021 | Food Preparation Workers | 0.73 |
|  | 35-9011 | Dining Room and Cafeteria Attendants and Bartender Helpers | 0.73 |
|  | 35-3021 | Combined Food Preparation and Serving Workers; Including Fast Food | 0.73 |
|  | 35-3031 | Waiters and Waitresses | 0.73 |
|  | 35-9031 | Hosts and Hostesses; Restaurant; Lounge; and Coffee Shop | 0.73 |
| Manufacturing | 51-2022 | Electrical and Electronic Equipment Assemblers | 0.6 |
|  | 51-2031 | Engine and Other Machine Assemblers | 0.6 |
|  | 51-4023 | Rolling Machine Setters; Operators; and Tenders; Metal and Plastic | 0.6 |
|  | 51-4031 | Cutting; Punching; and Press Machine Setters; Operators; and Tenders; Metal and Plastic | 0.6 |
|  | 51-2092 | Team Assemblers | 0.6 |
|  | 51-1011 | First-Line Supervisors of Production and Operating Workers | 0.6 |
|  | 51-9061 | Inspectors; Testers; Sorters; Samplers; and Weighers | 0.6 |
| Transportation and warehousing | 53-7062 | Laborers and Freight; Stock; and Material Movers; Hand | 0.6 |
|  | 53-3032 | Heavy and Tractor-Trailer Truck Drivers | 0.6 |
|  | 53-3033 | Light Truck or Delivery Services Drivers | 0.6 |
| Agriculture | 45-2090 | Miscellaneous Agricultural Workers | 0.57 |
|  | 45-2041 | Graders and Sorters; Agricultural Products | 0.57 |
|  | 45-4022 | Logging Equipment Operators | 0.57 |
| Retail trade | 41-2031 | Retail Salespersons | 0.53 |
|  | 41-2011 | Cashiers | 0.53 |
|  | 41-2021 | Counter and Rental Clerks | 0.53 |
| Mining | 47-5013 | Service Unit Operators; Oil; Gas; and Mining | 0.51 |
|  | 47-5041 | Continuous Mining Machine Operators | 0.51 |
|  | 47-5042 | Mine Cutting and Channeling Machine Operators | 0.51 |
| Other services | 39-9021 | Personal Care Aides | 0.49 |
|  | 39-9011 | Childcare Workers | 0.49 |
|  | 39-5012 | Hairdressers; Hairstylists; and Cosmetologists | 0.49 |
| Construction | 47-2061 | Construction Laborers | 0.47 |
|  | 47-4011 | Construction and Building Inspectors | 0.47 |
|  | 47-4099 | Construction and Related Workers; All Other | 0.47 |
| Utilities | 43-5081 | Stock Clerks and Order Fillers | 0.44 |
|  | 43-5071 | Shipping; Receiving; and Traffic Clerks | 0.44 |
|  | 43-5052 | Postal Service Mail Carriers | 0.44 |
| Wholesale trade | 13-1023 | Purchasing Agents; Except Wholesale; Retail; and Farm Products | 0.44 |
|  | 13-1022 | Wholesale and Retail Buyers; Except Farm Products | 0.44 |
|  | 13-1021 | Buyers and Purchasing Agents; Farm Products | 0.44 |
| Finance and insurance | 13-2051 | Financial Analysts | 0.43 |
|  | 13-2099 | Financial Specialists; All Other | 0.43 |
|  | 13-2053 | Insurance Underwriters | 0.43 |
|  | 13-1032 | Insurance Appraisers; Auto Damage | 0.43 |
| Arts, entertainment, and recreation | 27-1024 | Graphic Designers | 0.41 |
|  | 27-3041 | Editors | 0.41 |
|  | 27-2012 | Producers and Directors | 0.41 |
| Real estate | 11-9141 | Property; Real Estate; and Community Association Managers | 0.4 |
|  | 41-9022 | Real Estate Sales Agents | 0.4 |
|  | 13-2021 | Appraisers and Assessors of Real Estate | 0.4 |
|  | 41-9021 | Real Estate Brokers | 0.4 |
| Administrative | 43-1011 | First-Line Supervisors of Office and Administrative Support Workers | 0.39 |
|  | 11-3011 | Administrative Services Managers | 0.39 |
|  | 43-6011 | Executive Secretaries and Executive Administrative Assistants | 0.39 |
| Health care and social assistances | 29-1141 | Registered Nurses | 0.36 |
|  | 29-2061 | Licensed Practical and Licensed Vocational Nurses | 0.36 |
|  | 29-1060 | Physicians and Surgeons | 0.36 |
| Information | 15-1100 | Computer Occupations; All Other | 0.36 |
|  | 15-1150 | Computer Support Specialists | 0.36 |
|  | 15-1132 | Software Developers; Applications | 0.36 |
| Professionals | 17-2051 | Civil Engineers | 0.35 |
|  | 17-2141 | Mechanical Engineers | 0.35 |
|  | 17-2071 | Electrical Engineers | 0.35 |
| Management | 11-3031 | Financial Managers | 0.35 |
|  | 11-1011 | Chief Executives | 0.35 |
|  | 11-3121 | Human Resources Managers | 0.35 |
| Educational services | 25-1000 | Post-secondary Teachers | 0.27 |
|  | 25-2021 | Elementary School Teachers; Except Special Education | 0.27 |
|  | 25-2031 | Secondary School Teachers; Except Special and Career/Technical Education | 0.27 |
