# Supplementary material for: Can digital skill protect against job displacement risk caused by artificial intelligence? Empirical evidence from 701 detailed occupations
Source: PLoS One. 2022 Nov 8;17(11):e0277280. doi: 10.1371/journal.pone.0277280 (PMC9642882; doi:10.1371/journal.pone.0277280)
Supplement: Attachment — Submitted filename: Response to Reviewers.docx [file pone.0277280.s004.docx]

Dear Editor,

We appreciate you and the reviewers for taking much time in reviewing our paper. We have carefully considered the comments and tried our best to respond and revise each one of them in the past few days.

We provide the point-by-point responses highlighted in red as following. Sincerely, we are looking forward to your further comments.

Kind regards,

Ni Chen, Zhi Li and Bo Tang

1. Response to Journal Requirements and Editor Comments:

Q1: Please ensure that your manuscript meets PLOS ONE's style requirements, including those for file naming.

**Response 1:** Thanks for the reminder. We have carefully read PLOS ONE's style requirements once again, and then we have checked the manuscript throughout to correct the inappropriate points. All changes have been marked so that the editor and reviewers could see and understand them straightly.

Q2: In order to meet journal requirements for reporting and reproducibility, at this time we request that you please update the Methods section to report the original source of the data and the methods used to collect it in sufficient detail for another researcher to access the same data in the same manner. Please ensure that you include a statement specifying whether the collection and analysis method complied with the terms and conditions of the data source.

**Response 2:** Thanks for the recommendations. We have tried to make a rearrangement in this section of “3.Methods”. And we have reported the original source of the data used in this article, namely the public website of the databases. In addition to make our data collection more transparent and reproducibility, we have reported our stages of the data collection, and have uploaded all the data used in this study for sharing as in the file “S1 Dataset”. [Lines 162-183]

Q3: Please review your reference list to ensure that it is complete and correct. If you have cited papers that have been retracted, please include the rationale for doing so in the manuscript text, or remove these references and replace them with relevant current references. Any changes to the reference list should be mentioned in the rebuttal letter that accompanies your revised manuscript. If you need to cite a retracted article, indicate the article’s retracted status in the References list and also include a citation and full reference for the retraction notice.

**Response 3:** Thanks for the reminder. We have double-checked and corrected the in-text citations and end-text references list as the reviewer requested. In this revised manuscript, we have cited several new references as following:

16.Zúniga-Gonzalez, C.A. (2021). The role of the mediator and the student in the new educational scenarios: COVID-19. E J Qual High Educ. 2021;12(2), 279– 294. doi: 10.22458/caes.v12i2.3730.

34.Burda MC, Mertens A. Estimating wage losses of displaced workers in Germany. Labour Econ. 2001;8(1):15-41. doi: 10.1016/s0927-5371(00)00022-1.

41.Jimenez G, et al. Digital health competencies for primary healthcare professionals: A scoping review. Int J Med Inform. 2020;143(104260):104260. doi: 10.1016/j.ijmedinf.2020.104260.

42.Pichler D, Stehrer R. Breaking Through the Digital Ceiling: ICT Skills and Labour Market Opportunities. The Vienna Institute for International Economic Studies. 2021. Available from: https://www.econstor.eu/handle/10419/240636

45.Stephany F. Closing the Digital Skill Gap : The Potential of Online Platform Data For Active Labour Markets Policies. Zenodo. 2022. Available from: https://zenodo.org/record/6684547/files/Stephany-%20Closing%20the%20Digital%20Skill%20Gap.pdf?download=1

46.Organisation for Economic Co-operation and Development. Designing Active Labour Market Policies for the Recovery. OECD Publishing; 2021.

47.Stephany F, Kässi O, Rani U, Lehdonvirta V. Online Labour Index 2020: New ways to measure the world’s remote freelancing market. Big Data Soc. 2021;8(2):205395172110432. doi: 10.1177/20539517211043240.

We have not cited any papers that have been retracted, and we have tried our best to correct any errors we could find. However, if there are still inaccuracies, please point them out and allow us to correct.

Q4: Additional Editor Comments that suggesting some reference to add in introduction, review literature.

**Response 4:** Thanks for the recommendation. We have seriously read the recommended literature and cited some of them that are most relevant to our research topic in this article.

2. Response to Reviewer 1 Comments

Q1: The authors clearly lay out the main hypotheses of the article, and detail the methodology used to test each of these.

**Response 1:** Thanks for the reviewer’s positive comment.

Q2: “I don’t know” was selected because labor statistics fall outside the reviewer’s area of expertise. However, it is noted that the authors' statistical methods and underlying literature are explained in detail, and that for added rigor, authors conducted additional analysis with alternative displacement risk measures to confirm robustness of results. Some of the statistical methods and rationale (e.g. robustness analyses) are described in the results section; it would be good to rearrange the sections so that all analytical methods are presented together. It may also be helpful to shorten some of the tables (e.g. table 4) to focus on the most-affected categories, and include the full list in supplements.

**Response 2:** Thanks for the recommendations. We have added one separate section named “3.3.Statistical Analysis” in “3.Methods” to describe all the statistical analysis methods used in this study, including fixed effects model, grouped regression method and moderation effect testing. And we have reported that the statistical software STATA 16.0 was employed for all the statistical analyses. [Lines 188-195]

Then, we have followed the reviewer's suggestion to shorten the Table 4 and Table 6. After careful consideration, we decided to retain the occupational categories with significant effects both on occupational wage and employment [Lines 241]. In addition, we presented only the alternative measurement of displacement risk by sectors [Lines 282]. The detailed full list of tables was placed in “supporting information” of this revised manuscript.

Q3: Key variables from data sources are included in tables, and publicly-available data sources have been listed.

**Response 3:** Thanks for the reviewer’s positive comment.

Q4: The introduction and literature review sections are clear and compelling, even to an audience with limited expertise in the fields of AI and labor. However, the discussion and conclusion sections were less grounded in practical implications of the research. Given the relevance of this paper, it would be good to strengthen these sections by expounding on some of the recommendations:

- For example, a key recommendation is the need to develop more digital skills, and it is stated that the potential impact of these “cannot be under-estimated”. From the literature reviewed, could you provide a couple of examples to illustrate the effect of digital skills on occupational impacts of displacement risk from AI, especially for the most affected sectors?

**Response 4.1:** Thanks for the recommendations. We have listed the examples of the “healthcare sector” and the “hospitality, retail and other services managers” occupation category, to illustrate the benefits of improving digital skills to protect workers in the two most-affected occupational domains by AI [Lines 322-330].

- Active labor market policies are another example of a recommendation that would be enhanced by data-based examples, even if drawn from a limited number/span of studies.

**Response 4.2:** Thanks for the recommendations. We have added some specific and data-based examples as the reviewer’s suggestion, one of which is the online platform data for ALMPs in recent years while playing a critical role in closing the digital skill gaps such as skill training, job searching and so on [Lines 340-348].

- Lack of individual level data prevents understanding of other social factors that may affect findings and recommendations, as highlighted in the discussion and conclusion sections. These factors might also contribute to heterogeneity. If such data is available for any of the sectors studied, a deeper dive into this aspect could help to illustrate the interplay between social factors and observed impacts of displacement risk on occupational wages and employment.

**Response 4.3:** Thanks for the comments and suggestions. It is a great idea that the analysis of the displacement risk on occupational wages and employment via individual level data. In the process of this study, we had thought about detailed analyzing the impact of AI on the individual among occupations as you mentioned. However, the issues may require a separate article to deal with, we had to give up going further on this direction. We believe that the individual level data could help us more understand the relationship of AI and labor market, the detailed analysis must be useful but cannot be explored within the limits of this paper. Therefore, we regard this issue as a limitation of our research and a gap expected to focus on in future research.

- It would be good to discuss the findings of the robustness analysis.

**Response 4.4:** We have tried to explain and discuss the results of the robustness tests [Lines 292-298]. The main ideas are as follows:

On the one hand, in the previous literature, other studies have also summarized the phenomenon of wage losses for displaced workers caused by technological progress and wage premiums arose from emerging skills has already happened among many sectors, which is consistent with our findings.

On the other hand, we used “job polarization” to explain why the effects of the robustness tests on occupational employment are not significant, may the reason be that job polarization presenting U-shaped employment distribution as well known, which makes the effect of displacement risk on occupational employment failing to show significance in the linear regression.

- Minor copy-edits in a few places are needed.

**Response 4.5:** We have checked our manuscript throughout and edited for grammar and syntax to try our best to make sure our points clear and easy to be understood.

3. Response to Reviewer 2 Comments

Reviewer #2: The author of the article presented showed his methodology and database and obtained results that compared his hypotheses. Specifically. The author found that the regression results demonstrate that displacement risk negatively impacted occupational wage and occupational employment;suggesting that AI has a greater impact on occupational wages in higher occupational level. Therefore, I believe that the information presented has an appropriate methodological support for its publication.

**Response:** Thanks for the reviewer’s positive comment and encouragement. The changing in jobs and occupations caused by AI, has been a hot topic around the world nowadays. Although many countries have been concerned about the labor employment issues caused by AI technology for a long time, there are still no one-size-fits-all measures to deal with the protection of labors' rights in technological development. We still need to discuss and study more deeply and extensively for better policies.
